# Observation of conformational dynamics in single light-harvesting proteins from cryptophyte algae

**DOI:** 10.1063/5.0095763

**Published:** 2022-07-19

**Authors:** Raymundo Moya, Audrey C. Norris, Leah C. Spangler, Gregory D. Scholes, Gabriela S. Schlau-Cohen

**Affiliations:** 1 Department of Chemistry, Massachusetts Institute of Technology, Cambridge, Massachusetts 02139, USA; 2 Department of Chemistry, Princeton University, Princeton, New Jersey 08544, USA

## Abstract

Photosynthetic organisms use pigment–protein complexes to capture the sunlight that powers most life on earth. Within these complexes, the position of the embedded pigments is all optimized for light harvesting. At the same time, the protein scaffold undergoes thermal fluctuations that vary the structure, and, thus, photophysics, of the complexes. While these variations are averaged out in ensemble measurements, single-molecule spectroscopy provides the ability to probe these conformational changes. We used single-molecule fluorescence spectroscopy to identify the photophysical substates reflective of distinct conformations and the associated conformational dynamics in phycoerythrin 545 (PE545), a pigment–protein complex from cryptophyte algae. Rapid switching between photophysical states was observed, indicating that ensemble measurements average over a conformational equilibrium. A highly quenched conformation was also identified, and its population increased under high light. This discovery establishes that PE545 has the characteristics to serve as a photoprotective site. Finally, unlike homologous proteins from the evolutionarily related cyanobacteria and red algae, quenching was not observed upon photobleaching, which may allow for robust photophysics without the need for rapid repair or replacement machinery. Collectively, these observations establish the presence of a rich and robust set of conformational states of PE545. Cryptophytes exhibit particularly diverse energetics owing to the variety of microenvironments in which they survive, and the conformational states and dynamics reported here may provide photophysical flexibility that contributes to their remarkable ability to flourish under diverse conditions.

## I. INTRODUCTION

Cryptophytes are microalgae that are ubiquitous in diverse aquatic environments.^1^ They are thought to have originated from multiple endosymbiotic events in which cyanobacteria were engulfed by red algae and subsequently eventually evolved into the current organism, which is essentially a chloroplast.^2^ In contrast to most photosynthetic organisms in which the light-harvesting apparatuses are organized by membranes or linker proteins, the light-harvesting proteins of cryptophytes are densely packed within the luminal space without any higher order structure,^3–6^ leading to open questions about access by the protein machinery required for regulation and repair, and, thus, how cryptophyte light-harvesting proteins are robust under high light. The diverse environments where cryptophytes flourish have evolved a set of proteins with light-harvesting properties tuned to capture sunlight in different ecological niches.^2^ The light-harvesting proteins, known as phycobiliproteins (PBPs), contain phycobilin pigments covalently attached to a protein backbone with variations in the pigment identity, their position within the protein scaffold, and the structure of the scaffold itself.^3–7^ Although the overall structure and photophysics of several PBPs have been characterized, structural fluctuations are typical for a protein at physiological temperatures, and the impact of these fluctuations on the photophysics, if any, has not been investigated. Furthermore, how this photophysics varies under different conditions, such as high light, has not been determined.

The essential function of the PBPs is to capture sunlight and transfer the photoenergy to the reaction center, which initiates the biochemical reactions of photosynthesis. Energy transfer and other photophysical pathways depend sensitively on the positions of the pigments and the surrounding scaffold. As a result, structural fluctuations are expected to vary the energy transfer dynamics, yet ensemble measurements average over these fluctuations, obfuscating the conformational heterogeneity. Single-molecule methods have, therefore, emerged as a powerful tool to investigate heterogeneous sub-populations and reveal underlying conformational states and dynamics.^7–13^ The advent of multi-parameter single-molecule techniques that simultaneously measure variables, such as fluorescence intensity, lifetime, spectra, and polarization, has revealed a rich and dynamic energy landscape with different functional conformations.^14–18^ In cyanobacteria and red algae, the PBPs are organized into large rod-like structures, known as the phycobilisome.^2,19^ Previous single-molecule experiments on isolated PBPs from cyanobacteria and red algae and on intact phycobilisomes have found a wealth of heterogeneous behaviors including quenching charge transfer states,^20^ interconversion between functional states,^14,21^ and even long-lived (ms–s) quenched states of the light-harvesting apparatuses.^20,22,23^

**FIG. 1.**
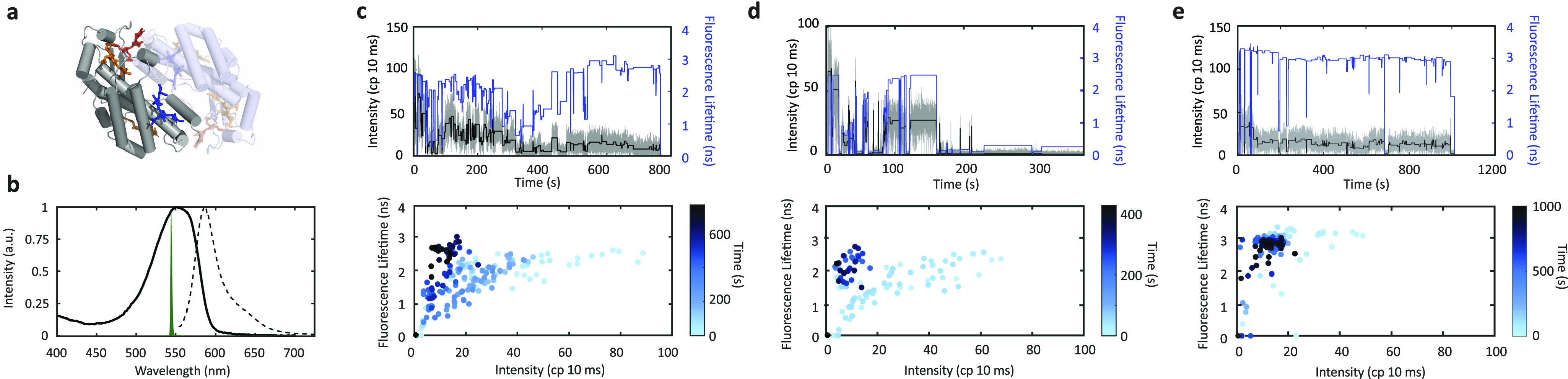
Single-molecule spectroscopy of PE545. (a) Structure of dimeric PE545 (PDB 1QGW) with monomer subunits in gray and faded blue and pigments in orange and dark blue. (b) Absorption (solid) and emission (dashed) spectra with the excitation laser overlaid in green. [(c), (d), (e); top] Representative single-molecule traces of the fluorescence intensity (gray) with the average for each period of constant intensity (black) and the concomitant fluorescence lifetime (blue). [(c), (d), (e); bottom] Corresponding scatter plots of the intensity-lifetime values for each period of constant intensity from the traces on the top row in (c), (d), and (e). The colormap indicates the observation time at which the value was detected.

One of the most widely studied cryptophyte PBPs is phycoerythrin 545 (PE545), so-called for its absorption maximum at ∼545 nm. The structure of PE545 is dimeric [Fig. 1(a)], with a heterodimer construction of peptide subunits known as *α* and *β*, as seen in other PBPs.^1^ Typically, there are multiple polypeptide variants of *α*, but the pigment composition and fluorescence properties of the variants are identical.^24^ Each monomer of PE545 binds four pigments. A dihydrobiliverdin (DBV) pigment (blue in 1a) is positioned at the distal end and is thought to be the lowest energy pigment, and thus the terminal emitter. Three phycoerythrobilin (PEB) pigments (orange/red in 1a) are bound within the alpha subunit.^25–27^ The highest energy PEB pigment is positioned at the monomer–monomer interface, which leads to a strong coupling upon dimerization and an exciton delocalized across the monomers. The strong coupling drives rapid energy transfer across the monomer–monomer interface and, thus, energetic equilibration throughout the dimer.^26–28^

In this work, we report single-molecule fluorescence measurements of PE545 from *Rhodomonas minuta* strain CPCC344, which is the first single-molecule study of a cryptophyte protein. These measurements revealed unquenched, quenched, and highly quenched (off) conformations and rapid transitions between the conformations, establishing the presence of conformational dynamics that influence the photophysics. The population of the off conformation increases with excitation fluence, which reflects the presence of non-radiative pathways to dissipate excess energy under high light. In contrast to other PBPs, photodegraded pigments do not quench the other pigments, ensuring robust light harvesting even in the absence of repair mechanisms. These results reveal heterogeneous yet robust behaviors that may contribute to the ability of cryptophyte proteins to capture sunlight under diverse conditions.

## II. METHODS

### A. PE545 purification, characterization, and sample preparation

Cryptophyte *Rhodomonas minuta* strain CPCC344 was cultivated and harvested, and the phycobiliproteins were isolated by the usual procedure.^25^ Following harvesting, the phycobiliproteins were isolated from cellular debris using one freeze/thaw cycle resulting in a protein rich supernatant. The phycobiliproteins were further purified using successive ammonium sulfate precipitation (40%, 55%, and 80%) followed by collection using ultracentrifugation for 25 minutes at 35000 *x* g. Residual ammonium sulfate was removed from the final phycobiliprotein solution by dialysis against 25 mM phosphate buffer, pH 6.8.

Solutions of purified PE545 complexes at ∼10 *µ*M were stored at −80 °C. The solutions were thawed over ice immediately prior to experiments and diluted in a 25 mM phosphate buffer at pH 6.6.200 *μ*l of the sample was added to a quartz 10 × 2 mm^2^ cuvette for steady-state measurements. Fluorescence spectra were measured using a Varian Cary Eclipse and absorbance spectra were measured in an Agilent Cary 5000 spectrometer.

### B. Single-molecule fluorescence measurements

The purified PE545 solutions were thawed over ice immediately prior to experiments and diluted to ∼ 500 pM in a 25 mM phosphate buffer containing 1% polyvinyl alcohol. An enzymatic oxygen-scavenging system was added to the solution at final concentrations of 25 nM protocatechuate-3,4-dioxygenase and 2.5 mM protocatechuic acid.^29,30^ 50 *μ*l of the solution was spin-coated onto glass coverslips where the determined concentration allowed for about 1 molecule per square micrometer.

Single-molecule fluorescence measurements were performed in a home-built confocal microscope. A fiber laser (FemtoFiber pro, Toptica; 130 fs pulse duration, 80 MHz repetition rate) was tuned to 545 nm and set to an excitation power of 10 and 30 nW before the microscope (120 and 360 nJ/cm^2^ at the sample plane, respectively, assuming a Gaussian beam). Sample excitation and fluorescence collection were accomplished by the same oil-immersion objective (UPLSAPO100XO, Olympus, NA 1.4). The fluorescence signal was isolated using a dichroic mirror (ZT568rdc-UF3, Chroma) before the objective and a bandpass filter (FF01-600/52-25, Semrock). The signal was detected using an avalanche photodiode (SPCM-AQRH-15, Excelitas), and photon arrival times were recorded using a time-correlated single photon counting module (TimeTagger20, Swabian Instruments). The instrument response function was measured from scattered light to be 380 ps (full width at half maximum). 170 complexes were measured at 10 nW and 89 complexes at 30 nW.

### C. Analysis of single-molecule data

The detected fluorescence photon stream was analyzed to determine the fluorescence intensity and fluorescence lifetime. Intensity levels were identified using the change-point-finding method developed by Watkins and Yang.^31^ The photons collected within each intensity level were then histogramed to construct a fluorescence decay trace. Each decay was fit to an exponential function convolved with the instrument response function using maximum likelihood estimation to incorporate the effect of Poissonian noise as described in previous work.^32^ Experimental fluorescence decays and their fits are shown in Fig. S1 of the supplementary material. To check for lifetime fluctuations within states identified through the change-point analysis, photons within a state were binned by every 1000 photons and no significant fluctuations were observed. These lifetime fluctuations are shown in Fig. S2 of the supplementary material. Two-dimensional histograms of different intensity and lifetime parameters were constructed, and a two-dimensional kernel density estimation algorithm was used to better visualize the distributions.

## III. RESULTS AND DISCUSSION

The absorption and fluorescence spectra of PE545 are shown in Fig. 1(b) overlaid with the excitation laser spectrum. At the low concentrations required for single-molecule investigation, the dimers are expected to have dissociated into monomer subunits.^21,33,34^ In order to confirm the monomer has similar emissive properties to the physiological dimer, we performed concentration-dependent measurements of the emission spectra and lifetime ranging from concentrations at which PE545 is monomeric (Fig. S3 of the supplementary material) up to concentrations at which it is primarily dimeric.^35^ Similar spectra and lifetime decay curves were observed for all concentrations (Figs. S4 and S5 of the supplementary material). Consistently, previous work showed similar steady-state spectra for the monomer and dimer.^35^ These results suggest that the monomer maintains the secondary structure and properties of the dimer.

Single-molecule fluorescence intensity and lifetime time traces are shown in Figs. 1(c)–1(e) (top), which capture representative behaviors of the individual complexes. Rapid transitions between different values of intensity and lifetime were present. To visualize the evolution associated with these transitions, fluorescence intensity-lifetime scatter plots were constructed for both representative traces. Each period of constant intensity is plotted with its concomitant lifetime in Figs. 1(c)–1(e) (bottom). Qualitatively, two types of behaviors were observed. At early observation times (light blues), the complexes exhibited correlated changes in fluorescence intensity and lifetime along with decreasing intensity as similar lifetime values were maintained, generating a bounded-exponential-type curve. At later observation times, i.e., after extended illumination (dark blues), the fluorescence intensity was significantly reduced with both long lifetime values [Figs. 1(c)–1(e), bottom] and short ones [Fig. 1(c), bottom], most likely due to photobleached pigments no longer contributing to the absorption cross section.

### A. Emissive states of PE545

**FIG. 2.**
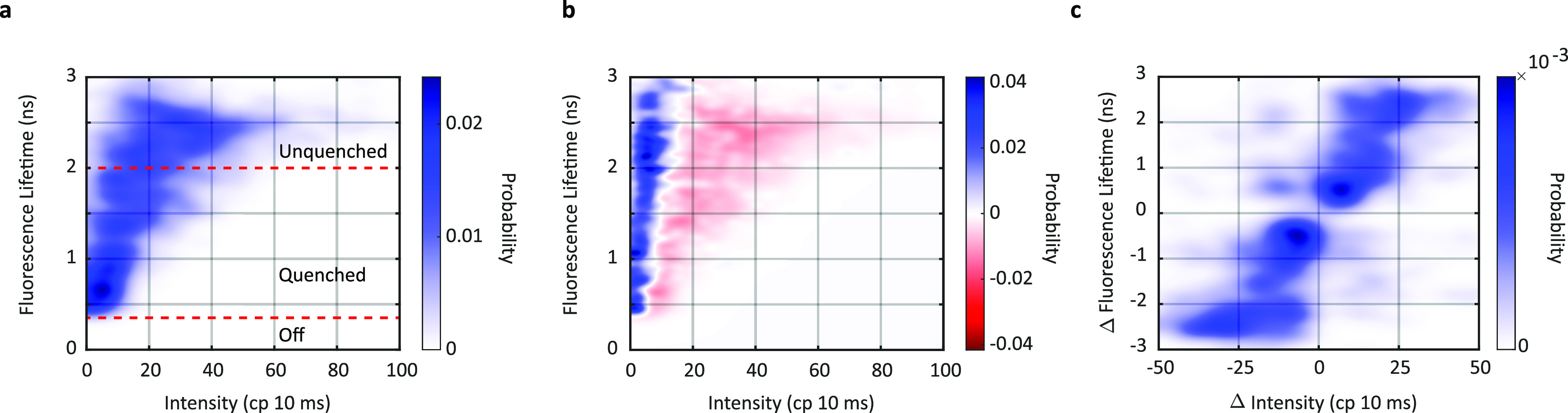
Two-dimensional histograms of intensity and lifetime. (a) Fluorescence intensity-lifetime probability distribution from measurements of single PE545 (N = 170 molecules, M = 1835 intensity levels) at low power (10 nW) and early times. (b) Difference-density plot between the estimated probability distributions for late times (final 25%) and early times (initial 25%) of the single-molecule trace. (c) Change in fluorescence intensity-lifetime distributions associated with each change of intensity for PE545 at low power (10 nW) and early times.

Two-dimensional histograms were constructed from the concomitantly measured fluorescence intensity and lifetime values for all the PE545 complexes [Fig. 2(a), Fig. S7]. These histograms show the overall distributions of different photophysical behaviors within the heterogeneous ensemble. The profile of the distribution is similar to PBPs from cyanobacteria, which also exhibit a range of unquenched (high intensity, long fluorescence lifetime) and quenched (low intensity, shorter fluorescence lifetime) states.^21,36,37^ The distributions can be approximately divided into three states: (1) an unquenched state with an intensity of ∼35 cp 10 ms and a fluorescence lifetime of ∼2.45 ns; the unquenched state is the predominant population prior to extended illumination (Fig. S6 of the supplementary material), which is captured as all measurements above 2 ns; (2) a quenched state with an intensity of ∼20 cp 10 ms and a fluorescence lifetime of ∼1.5 ns; and (3) an off, or fully quenched, state with a fluorescence intensity of ∼5 cp 10 ms and a fluorescence lifetime of <0.4 ns. Lifetime values below the 380 ps of the instrument response function are generally dominated by the instantaneous response of the background emission, leading to the assignment of the off state. These different emissive states are generally thought to arise from differences in the pigment photophysics and/or protein conformations.^7,20,21^

The presence of quenched states establishes that PE545 can exist in quenched conformations. Present theories suggest that quenching can be functionally ingrained into the intrinsic photophysics of attached pigments. Proposed quenching mechanisms include, but are not limited to, inter- and intramolecular charge transfer states,^38,39^ radical cations,^37,40^ and photochemical processes.^21,37^ These quenched conformations, as well as the quenched conformations of plant light-harvesting proteins, exhibited photoprotective behaviors, which is the safe dissipation of excess energy by oxygenic photosynthetic organisms under high light conditions. Therefore, PBPs of cryptophyte algae exhibit a similar ability to adopt quenched conformations as other photosynthetic proteins, although ensemble measurements indicate they are not directly involved in photoprotection.^41^

### B. Characterization of photodegradation

To isolate the influence of photodegradation on the observed distributions, the single-molecule traces were divided based on observation time. The intensity and lifetime values were split into three groups: early times [the first 25% of the experimental trace; Fig. 2(a)], intermediate times (25%–75% of the trace; Fig. S7b), and late times (75%–100% of the trace; Fig. S7c). To determine the changes upon illumination, the difference between the late and early intensity-lifetime histograms was calculated. The resultant difference-density plot is shown in Fig. 2(b) (high power difference-density plots are shown in Fig. S9a), which directly illustrate the photoinduced changes in the populations of the photophysical states. Consistent with the qualitative observations in Figs. 1(c)–1(e) (bottom), the population at higher intensities decreases (red regions) while the population at lower intensities increases (blue regions), reflecting an overall decrease in intensity. The large slope of the nodal line between the blue and red regions indicates a small shift toward a longer lifetime with an overall similar distribution of values. These results show that both unquenched and quenched conformations in terms of fluorescence lifetime are maintained through the photodegradation process, which decreases the intensity.

The DBV pigment is thought to be the lowest in energy and so is likely to photodegrade first due to the increased amount of time the excitation localizes on this site. The PEB 82 pigment [orange, Fig. 1(a)] is thought to be the next lowest in energy and so likely also photodegrades relatively quickly. Therefore, PEB158 and PEB50/61 [orange and red, respectively Fig. 1(a)] likely dominate the later portion of the measurement time. Under these assumptions, the similar lifetime distributions at the beginning and end of the measurement time [Fig. 2(a)] suggest minimal lifetime heterogeneity among the pigments despite their different binding sites.

### C. Transitions between emissive states

To analyze the ability of PE545 to switch between emissive states, the change in intensity (Δ Intensity) and lifetime (Δ Lifetime) was calculated for each change in intensity levels. The values for all the transitions were used to construct the histogram as shown in Fig. 2(c) (high power difference-density plots are shown in Fig. S9b), which illustrate the distributions of dynamics between photophysical states. In this plot, the upper right quadrant contains transitions to brighter and longer lifetime states while the bottom left quadrant contains transitions to dimmer and shorter lifetime states, i.e., shifts into more quenched conformations. The lack of a significant population in the other two quadrants reflects correlated changes for intensity and lifetime, as expected from changes in the rate of non-radiative decay.

In Fig. 2(c), the features can be described as four peaks. The peaks are understood as correlated transitions in intensity and lifetime reflected diagonally across the origin. The uppermost peak (∼25 cp 10 ms, ∼2.5 ns) corresponds to transitions from the off to the unquenched state whereas the lowermost peak (∼−25 cp 10 ms, ∼−2.5 ns) corresponds to transitions from the unquenched to the off state. The other positive peak, which is a broad feature <25 cp 10 ms and <2 ns, contains both transitions from the off to the quenched state and from the quenched to the unquenched state. Similarly, the other below negative peak, which is also a broad feature <25 cp 10 ms and <2 ns, contains both transitions from the quenched to the off state and from the unquenched to the quenched state. For both features, the high intensity region near the origin is due to transitions within the states.

Therefore, three types of transitions were observed: (1) dynamics between emissive (unquenched, quenched) states, which likely reflects conformational changes of PE545; (2) blinking, which is the reversible switching into an off state from the emissive states; and (3) photobleaching, which is an intensity decrease within a lifetime state. We ascribe a transition to lower intensity to photodegradation due to the overall intensity decrease upon illumination, as described above and illustrated in Fig. 2(b).

Overall, the presence of a significant population across the diagonal line indicates a high degree of connectivity between the states, i.e., transitions occur between all states. Furthermore, the similar population on both sides of the origin reveals that the majority of these transitions are reversible. In contrast to other PBPs where degradation into lower intensity and shorter lifetime states was primarily observed, PE545 exhibits reversible dynamics, likely reflecting additional conformational dynamics for this protein.

### D. Kinetics of transitions between emissive states

**FIG. 3.**
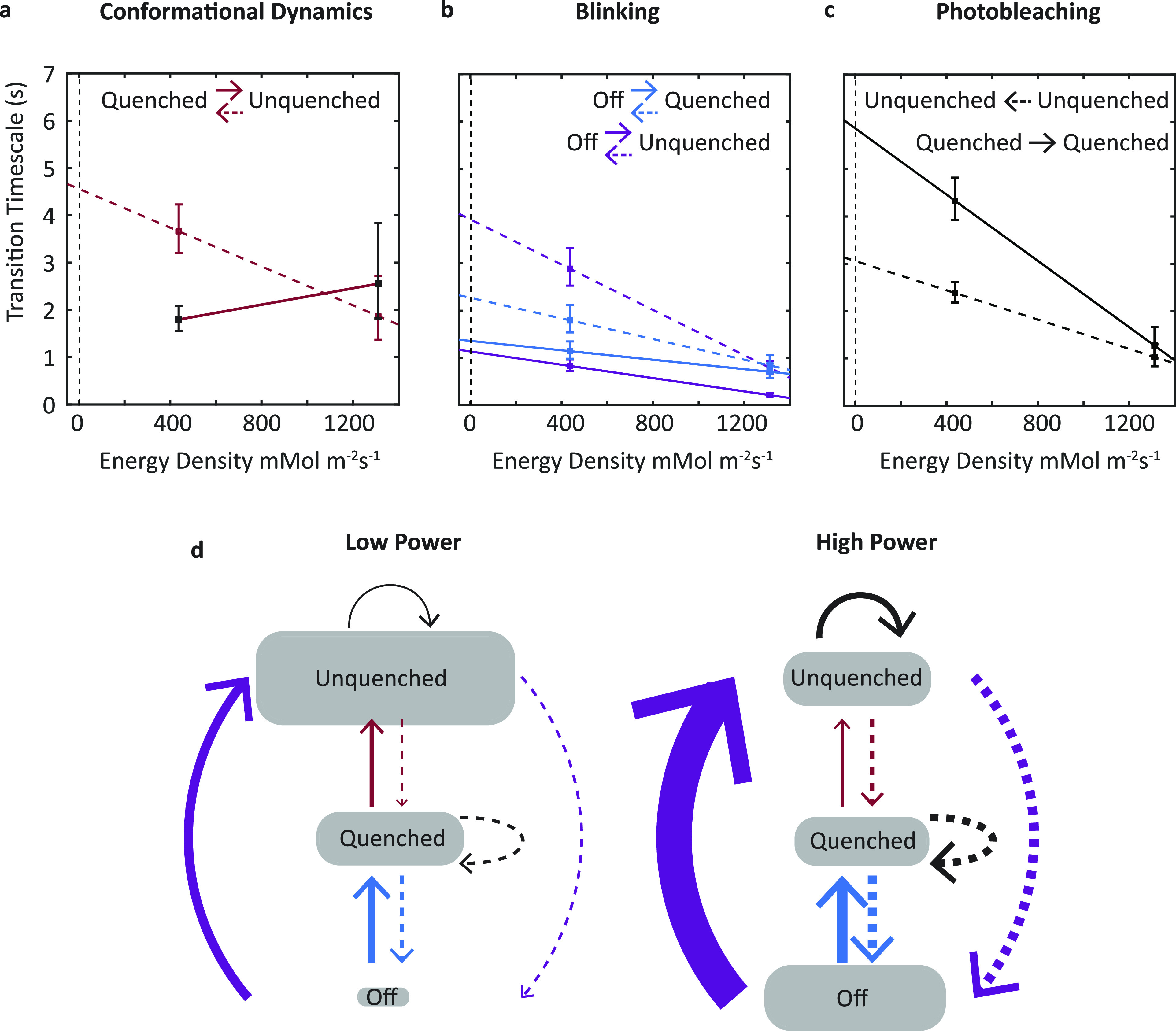
Emissive states and dynamics. (a) Timescales for transitions between the unquenched and quenched states. (b) Timescales for transitions into and out of the off state, i.e., blinking. The power dependence of the transitions out of the off state indicates the protein remains photoactive. (c) Timescales for transitions within a single emitting state, i.e., intensity decreases without a lifetime change, which predominantly arise from pigment photobleaching. Error bars show 95% confidence intervals and the dashed vertical line at 2 mMol m^−2^ s^−1^ indicates the energy density of sunlight. (d) Schematic indicating the emissive states and their transitions for the two power conditions. The relative population fractions in each state are indicated by the size of oval and the relative timescales of the transitions are indicated by the thickness of the arrows.

To further characterize the dynamics associated with the transitions observed in Fig. 2(c), the timescales, i.e., the inverse of the rates, were quantified for each transition. The dwell times, which are the duration of the intensity level prior to a transition, were calculated for all transitions.^8^ For each combination of initial and final lifetime states [Fig. 2(a)], a histogram of dwell times was constructed and fit to an exponential probability distribution using maximum likelihood estimation. An example histogram with the corresponding fit is shown in Figure S10 and the extracted timescales for all transitions between the lifetime states are plotted in Figs. 3(a)–3(c) and shown along with the relative populations in the schematics in Figs. 3(d) and 3(e). All transitions were found to occur on timescales of one to five seconds.

The timescale calculations shown in Fig. 3 were limited to the early times (first 25%) of the single-molecule traces to minimize the influence from photodegraded PE545 on the extracted rates. The timescales for early, intermediate, and late times are plotted in Fig. S11. The timescales all slowed as a function of observation time, likely due to photobleaching of pigments that removed their contribution to the system and thereby reduced the number of pathways available.

The single-molecule measurements were performed at two different powers of the excitation laser, 10 mW (400 mM m^−2^ s^−1^) and 30 mW (1200 mM m^−2^ s^−1^), and the timescales were calculated for both datasets. To determine the power dependence of all types of transitions, the values at both powers were fit to a linear function. Analysis of these fits enables photoinduced and thermal dynamics to be distinguished, as they give rise to negative and near-zero slopes, respectively. The photoinduced dynamics can either be a process accessible from the excited-state surface or a process for which the enormous energy of the photon (∼100 k_B_T) overcomes an activation barrier. However, the data cannot distinguish between these mechanisms. Linear scaling was assumed as the simplest possible model of intensity dependence, although full or partial nonlinear scaling of the rates is also a possibility. Investigations of these effects were precluded by the limited range of powers in the single-molecule measurements. Under the assumption of linearity, the fit functions were used to extrapolate the dynamics to the energy density of sunlight.^20^ From this extrapolation, all transitions are predicted to occur on timescales of one to six seconds, indicating that the dynamics observed here are relatively frequent under physiological conditions.

***Conformational dynamics***. For the dynamics between the unquenched and quenched states [Fig. 3(a)], the unquenched to quenched transition becomes approximately twice as frequent (∼2–∼4 sec) at higher powers, leading to a steep slope and, thus, indicating a photoinduced process. In contrast, no significant change was found for the ∼2.5 sec timescale of the quenched to unquenched transition, leading to a near-horizontal line and, thus, indicating a thermal process. Even though one of these rates is photoinduced, the dependence is such that the unquenched and quenched conformations, and thus their associated dynamics, are both present under physiological conditions. The mechanism behind the formation of a quenched state can be conformational or photochemical in origin. Recent work in PBPs from cyanobacteria has suggested that photoinduced conformational changes lead to quenching by altering the interactions between pigments and neighboring amino acids. In this model, subsequent photoexcitations then lead to the presence of charge transfer states, which are red-shifted with low fluorescence emission.^38,39^ Consistent with this picture, the addition of chemical cross-linkers that stabilize the protein structure led to slower transitions between the unquenched and quenched states.^20^ Based on these results, we assign the dynamics between the unquenched and quenched states as likely due to protein conformational changes. Alternatively, quenching by photoinduced radical cations has been proposed^36,42^ and observed for ultraviolet excitation.^43^ Finally, annihilation between singlet excitations or between singlet and triplet excitations can also form quenchers.^42^ These alternative mechanisms may also be partially or fully responsible for the observed states and dynamics.

For the blinking dynamics [Fig. 3(b)], transitions between the unquenched and off states become approximately four times as frequent at higher powers whereas the transitions between the quenched and off states become approximately three times as frequent. The blinking timescales all have a steep slope as a function of energy density, and thus, these dynamics are all photoinduced. Notably, the photoinduced nature of the transitions from the off state to the quenched and unquenched states suggests that the off state is actually a “strongly quenched” state as it retains its photoactivity. Therefore, these transitions are likely conformational in nature as well.

Overall, the changes to the conformational and blinking dynamics at higher power lead to a re-equilibration of the population from the unquenched state into the off (or strongly quenched) state. The shift into quenched states has the potential to be related to biological processes, such as repair and photoprotection. *In vivo* PE545 is found as a dimer, and so the protein conformations and dynamics within monomeric PE545 could be shifted by the interactions along the monomer–monomer interface. However, molecular dynamics simulations showed that differences between the two protomers predicted from the structure are erased by thermal fluctuations of the protein.^27^ First, while cyanobacteria and red algae can quickly repair or replace damaged PBPs, experiments have shown that PBPs within cryptophytes are not as mobile as the phycobilisome.^44^ The switch to more quenched states could allow for the safe dissipation of excess excitation energy preventing photodamage and making replacement mechanisms superfluous. Second, photoprotective pathways to dissipate excess energy under high light, known as non-photochemical quenching (NPQ), have been established in cryptophytes.^45–48^ In *Rhodomonas salina*, a related species to the one studied here, NPQ was found to occur in the membrane light-harvesting complexes, not in phycobiliproteins.^47^ However, in cyanobacteria, which share an evolutionary ancestor with cryptophytes, NPQ has been found to occur in both the membrane light-harvesting complexes and the phycobiliproteins. Depending on the species, either one or both of the mechanisms of NPQ are present.^49,50^ Therefore, while phycobiliprotein-based NPQ has not yet been identified in cryptophytes, future studies of other species, such as the *Rhodomonas minuta* investigated here, may find such a process, potentially mediated by the PE545 conformational dynamics shown in Fig. 3. The conformational and blinking dynamics occur on a seconds timescale and, thus, could mediate fast photoprotective responses on tens of seconds to minutes timescale.

***Photobleaching dynamics***. The photobleaching dynamics [Fig. 3(c)], which are intensity decreases within the lifetime states, become much more frequent at higher powers, indicating the expected photoinduced behavior for this process. The transitions in the unquenched state increase by a factor of 3.4, while the transitions in the quenched state increase by a factor of 2.3. The ratio of the lifetimes (2.5–1.5 ns, or 1.7) is approximately the same as the ratio of the increase in transitions (3.4–2.3, or 1.5), which indicates that photobleaching scales with the time spent in the excited state. Consistently, the blinking dynamics are faster for the unquenched to off transition than the quenched to off one, indicating a similar scaling may be present. These dynamics are assigned to the irreversible photobleaching, i.e., loss of photoactivity of individual pigments. The most likely mechanism behind photobleaching is photoexcitation followed by intersystem crossing to a triplet state, which sensitizes triplet molecular oxygen to generate highly reactive singlet oxygen species.^42^ Alternatively, annihilation or photoexcitation of long-lived triplets could play a role.^42^

Previous work on PBPs from cyanobacteria and red algae observed photodegradation through two processes: (1) an intensity decrease, assigned to pigment photobleaching as observed here, and (2) a correlated decrease in intensity and lifetime, assigned to photo-induced and irreversible conversion of pigments into quenchers.^21,36,37^ The second of these processes appears as a correlated decrease in intensity and lifetime upon formation of quenchers and was not observed in PE545, as shown through the similar lifetime distributions at early and late measurement times [Fig. 2(b)]. The lack of photobleaching induced quenching means that photodegradation for this complex has a smaller impact on photophysics than for other PBPs. Maintaining the photophysics of the intact complexes may be an evolutionary adaptation to the difficulty associated with specific targeting of the soluble PE545 proteins by repair or replacement machinery. Altogether, the single-molecule results imply a complex set of transitions in PE545 that involve thermal conformational dynamics, the ability to activate photoprotective quenching, and photophysics robust to photodegradation.

## IV. CONCLUSION

In this work, we used single-molecule fluorescence spectroscopy to identify emissive states and dynamics of the light-harvesting complex PE545 from cryptophyte algae. By simultaneously monitoring the fluorescence intensity and lifetime of single complexes, we can identify states and monitor the transitions between them. From these measurements, conformational dynamics were discovered as well as photodegradation pathways in which pigments are energetically removed from the coupled system. Finally, the transition timescales revealed a photo-induced bias toward quenched states, which could work to safely dissipate excess excitation energy.

The diverse environments that cryptophyte algae thrive in have led to a homologous protein with distinct energetics to efficiently harvest solar energy in each ecological niche. While early studies have characterized how larger structural changes affect spectra and ultrafast dynamics, these results provide a first look at the underlying conformational dynamics that have the potential to adapt to different light intensities without complex regulation and repair machinery. Future work on the homologous proteins may inform upon how pigment makeup, structure, and available dynamics have been optimized in these organisms.

## SUPPLEMENTARY MATERIAL

See the supplementary material for ensemble fluorescence methods and additional analysis.

## Data Availability

The data that support the findings of this study are available from the corresponding author upon request.
